# Correction: Ultra-low concentration gel polymer electrolytes realize stable and low-temperature lithium–organic batteries

**DOI:** 10.1039/d6sc90039a

**Published:** 2026-02-19

**Authors:** Mengjie Li, Hang Liu, Hai Su, Zehui Fan, Yuansheng Liu, Jixing Yang, Wei Zhu, Qinghao Chen, Yunhua Xu

**Affiliations:** a School of Materials Science and Engineering, Key Laboratory of Advanced Ceramics and Machining Technology (Ministry of Education), Tianjin University Tianjin 300072 China jackieyang@tju.edu.cn yunhua.xu@tju.edu.cn; b Institute of Technology for Carbon Neutrality, Shenzhen Institutes of Advanced Technology, Chinese Academy of Sciences Shenzhen 518055 China

## Abstract

Correction for ‘Ultra-low concentration gel polymer electrolytes realize stable and low-temperature lithium–organic batteries’ by Mengjie Li *et al.*, *Chem. Sci.*, 2026, https://doi.org/10.1039/d5sc09108j.

The email address of one of the corresponding authors, Jixing Yang (jackieyang@tju.edu.cn), was omitted from the published article. The correct affiliations, including email addresses, are as shown here.

This correction therefore includes the addition of the email address of one of the corresponding authors, Jixing Yang (jackieyang@tju.edu.cn).

The Royal Society of Chemistry apologises for these errors and any consequent inconvenience to authors and readers.

